# TRIM16 overexpression induces apoptosis through activation of caspase-2 in cancer cells

**DOI:** 10.1007/s10495-013-0813-y

**Published:** 2013-02-13

**Authors:** Patrick Y. Kim, Aldwin Suryo Rahmanto, Owen Tan, Murray D. Norris, Michelle Haber, Glenn M. Marshall, Belamy B. Cheung

**Affiliations:** 1 Children’s Cancer Institute Australia for Medical Research, Randwick, NSW 2031 Australia; 2 Centre for Children’s Cancer and Blood Disorders, Sydney Children’s Hospital, Randwick, NSW 2031 Australia

**Keywords:** Apoptosis, TRIM16, Caspase-2, Mitochondrial depolarisation, Cytochrome *c*

## Abstract

TRIM16 exhibits tumour suppressor functions by interacting with cytoplasmic vimentin and nuclear E2F1 proteins in neuroblastoma and squamous cell carcinoma cells, reducing cell migration and replication. Reduced TRIM16 expression in a range of human primary malignant tissues correlates with increased malignant potential. TRIM16 also induces apoptosis in breast and lung cancer cells, by unknown mechanisms. Here we show that overexpression of TRIM16 induces apoptosis in human breast cancer (MCF7) and neuroblastoma (BE(2)-C) cells, but not in non-malignant HEK293 cells. TRIM16 increased procaspase-2 protein levels in MCF7 and induced caspase-2 activity in both MCF7 and BE(2)-C cells. We show that TRIM16 and caspase-2 proteins directly interact in both MCF7 and BE(2)-C cells and co-localise in MCF7 cells. Most importantly, the induction of caspase-2 activity is required for TRIM16 to initiate apoptosis. Our data suggest a novel mechanism by which TRIM16 can promote apoptosis by directly modulating caspase-2 activity.

## Introduction

The tripartite motif or TRIM, family of proteins were originally described by its multi-domain design of three structurally distinct motifs, the RING finger zinc-binding domain, a B-box zinc-binding domain and the coiled-coil domain [1]. Presently, there are approximately 70 TRIM proteins which are structurally similar, yet involved in a diverse range of cellular processes including cell proliferation and differentiation, oncogenesis, apoptosis and retroviral replication [2, 3]. TRIM protein functions commonly relate to direct regulatory protein–protein interactions [4]. Since its identification in human mammary epithelial cells, several studies have documented the important physiological functions of the Tripartite Motif 16 (TRIM16) protein in cellular biology. While TRIM16 lacks the RING finger protein domain, it is comprised of two B-box domains that are followed by a coiled-coil region and a C-terminal, RFP/B30.2-like domain [5, 6]. A significant number of TRIM proteins positively or negatively regulate pathways associated with tumour progression and suppression, thus acting as oncogenes or tumour suppressors, respectively [4]. Some TRIMs, such as TRIM13, TRIM8, and TRIM32, function as tumour suppressor proteins through regulation of transcription and apoptosis [7–9]. While other TRIM proteins, such as TRIM27/RFP and TRIM24/TIF1α, function normally as tumour suppressor proteins, but acquire oncogenic activity when fused to kinases by tumour-associated chromosomal rearrangements [1].

TRIM proteins inhibit tumour development through multiple mechanisms, and one common pathway relates to the activation of apoptosis. A large network of interacting proteins mediates the highly orchestrated pathways of programmed cell death [10], and several TRIM proteins have been characterised as having regulatory roles in initiating and executing apoptosis. Overexpression of TRIM13 stabilises p53 and induces apoptosis [7], while TRIM27/RFP triggers apoptosis via stress-response kinases and caspases [1]. In addition to inducing apoptosis by activating tumour necrosis factor [8], TRIM32 enhances neural differentiation by acting as a co-activator of retinoid receptor, RAR*α*-mediated transcription which promotes neural differentiation [9]. Similar to these proteins, TRIM16 acts as a tumour suppressor protein through inhibitory effects on cell growth, migration, proliferation, as well as induction of apoptosis [11–14].

TRIM16 is also known as the estrogen-responsive B-box protein due to its original discovery as an estrogen-responsive protein in human mammary epithelial cells [6]. Further characterisation has implicated TRIM16 in an array of functions in human physiology. Estrogens and keratinocyte growth factor regulate mRNA expression levels of TRIM16 in human mammary epithelial cells and keratinocytes, respectively [5, 6]. Increased expression of TRIM16 induced the differentiation of keratinocytes [5]. In addition to these roles, TRIM16 interacts via its C-terminal RFP/B30.2-like domain with the components of the inflammasome and increases the secretion of IL-1β, enhancing innate immunity [15].

Like TRIM24/TIF1*α* and TRIM32, TRIM16 has been shown to suppress tumour progression through regulatory pathways involved in growth inhibition, migration, differentiation and apoptosis [12–14]. TRIM16 was identified as a key regulator of the retinoid anti-cancer signal in human neuroblastoma and breast cancer cell lines [12, 14]. TRIM16 enhanced and restored the growth inhibitory and anti-proliferative effects of retinoids through up-regulation of retinoid target genes, RARβ and CYP26A1 [11, 14]. TRIM16 protein expression in primary tissues from human neuroblastoma and squamous cell carcinoma of skin is decreased in the more malignant phenotype [12, 13]. Decreased cellular proliferation and migration of neuroblastoma and squamous cell carcinoma cell lines by directly interacting with and reducing protein stability of cytoplasmic Vimentin and nuclear E2F1, respectively [12, 13]. Most recently, we have demonstrated that TRIM16 can heterodimerize with other TRIM proteins and has E3 ubiquitin ligase activity [16].

Enforced overexpression of TRIM16 induces apoptosis in MB-MDA-231 breast and SK-MES-1 lung cancer cells [14], however, the exact mechanisms of TRIM16 involvement in the regulation of apoptosis remains unclear. In this study, we show that overexpression of TRIM16 induced apoptosis in malignant, but not non-malignant, cells, by binding to and activating caspase-2.

## Materials and methods

### Cell culture

BE(2)-C cell line was gifted by Dr. J. Biedler (Memorial Sloan–Kettering Cancer Center, New York). MCF7 and the human embryonic kidney 293 cells (HEK 293) were purchased from the American Type Culture Collection. All cells were cultured at 37 °C in 5 % CO_2_ as adherent monolayer in Dulbecco modified Eagle medium (Life Technologies) supplemented with l-glutamine and 10 % foetal calf serum.

### Transient transfection of plasmid DNA or siRNA

Full-length human TRIM16 plasmid DNA as described previously [11], was used for overexpression and transient transfections. siRNAs specific to TRIM16 (Dharmacon) and caspase-2 (Dharmacon) were used for knock-down. pcDNA3.1-Myc/His EV plasmid (Life technologies) and On-Target Plus scramble RNA (Dharmacon) were used as transient transfection controls. Sequences for TRIM16 siRNA were ACCUGCAUGGUGAAUUACUUU and caspase-2 siRNA were GCCUUGCACUCCUGAAUUU.

### Trypan blue exclusion cell viability assay

Human MCF7 breast cancer cells (1 × 10^6^ cells/flask) were transfected with either TRIM16-Myc/His or EV control and incubated for 24 and 48 h. At each time point the cells were harvested and mixed with trypan blue. Viable cells were counted on a haemocytometer.

### TUNEL apoptosis assay

TRIM16 overexpressing or EV transiently transfected (control) MCF7, BE(2)-C and HEK293 cells were stained with TUNEL TMR dye using the In Situ Cell Death Detection Kit (Roche) according to the manufacturer’s protocol. Samples were analysed using IF microscopy with a Zeiss Axiovert 200 M fluorescent microscope coupled to an AxioCamMR3 camera and driven by the Axio vision software. TUNEL positive cells were counted in each sample for quantification.

### Western immunoblot analysis and antibodies

Whole cell lysates were obtained with NP-40 cell lysis buffer (50 mM Tris–HCl pH 8.0, 150 mM NaCl, 1 % (v/v) IGEPAL). To isolate and separate cytosolic and mitochondrial proteins, the mitochondrial isolation kit (Thermo Scientific) was used according to the manufacturer’s protocol. Protein concentrations were measured with the BCA protein assay (Thermo Scientific). A final total of 20 μg whole cell protein extracts were loaded onto 4–20 % Criterion Tris–HCl gels (Bio-Rad) and then transferred onto nitrocellulose membranes for antibody detection.

Antibodies used for Western immunoblots were mouse monoclonal antibodies for Myc-tag; 1:4,000, (Cell Signalling Technologies) and GAPDH; 1:10,000, (Abcam). Rabbit polyclonal antibodies were for caspase-2; 1:500, (Abcam), cytochrome *c*; 1:1,000, (Cell Signalling Technologies), TRIM16; 1:4,000 (Bethyl Laboratories) and topoisomerase 1; 1:10,000 (Novus Biologicals).

### Immunoprecipitation assay

Protein lysates (200 μg) from TRIM16-Myc/His and EV (control) transiently transfected MCF7 cells were used for co-immunoprecipitation (co-IP) studies. Recombinant TRIM16-Myc/His was pulled-down from the sample using BcMag^®^ His Magentic beads (Bioclone Inc) according to the manufacturer’s protocol. Samples were analysed for interaction with Western immunoblots as described earlier.

### IF confocal microscopy

MCF7 cells (1 × 10^4^ cells/well) were grown in eight-well chamber slides and transiently transfected with TRIM16-Myc/His or EV (control) and incubated for 48 h. Cells were then fixed in 4 % (v/v) paraformaldehyde and the non-specific staining was blocked with 10 % (w/v) BSA. TRIM16-Myc/His was detected with mouse monoclonal Myc-tag antibody and caspase-2 was detected with rabbit polyclonal caspase-2 antibody. Samples were then incubated with Alexa Fluor 499 goat anti-mouse and Alexa Fluor 555 goat anti-rabbit. After the staining, the cells were mounted in ProLong Gold Antifade reagent with DAPI (Life Technologies). Images were obtained with the Olympus Fluoview FV1000.

### Caspase-2 activity assays

Activity of caspase-2 was measured in the whole protein lysates of TRIM16 and EV (control) transiently transfected MCF7 cells using the caspase-2 colourimetric assay kit (Abcam), following the manufacturer’s protocol. Active forms of caspase-2 were detected by Western immunoblots as described above. To inhibit the activity of caspase-2, cell samples were treated with, 20 μM Z-VDVAD-FMK, (BioVision).

### JC-1 mitochondrial membrane potential assay

The mitochondrial membrane potential of TRIM16-Myc/His and EV transiently transfected MCF7 cells was measured following the protocol of the MitoProbe™ JC-1 assay kit. Non-treated MCF7 cells served as controls for the assay. Mitochondrial depolarisation was measured by flow cytometry using the FACSCalibur.

### Statistical analysis

Statistical significance was evaluated using one-way ANOVA followed by Bonferroni’s multiple comparison post-test. *P* values <0.05 were considered statistical significant. GraphPad Prism 5 (La Jolla, CA) was used for statistical analysis.

## Results

TRIM16 induces apoptosis in MCF7 breast cancer, and BE(2)-C neuroblastoma cells but not in the non-malignant HEK293 cells.

**Fig. 1:**
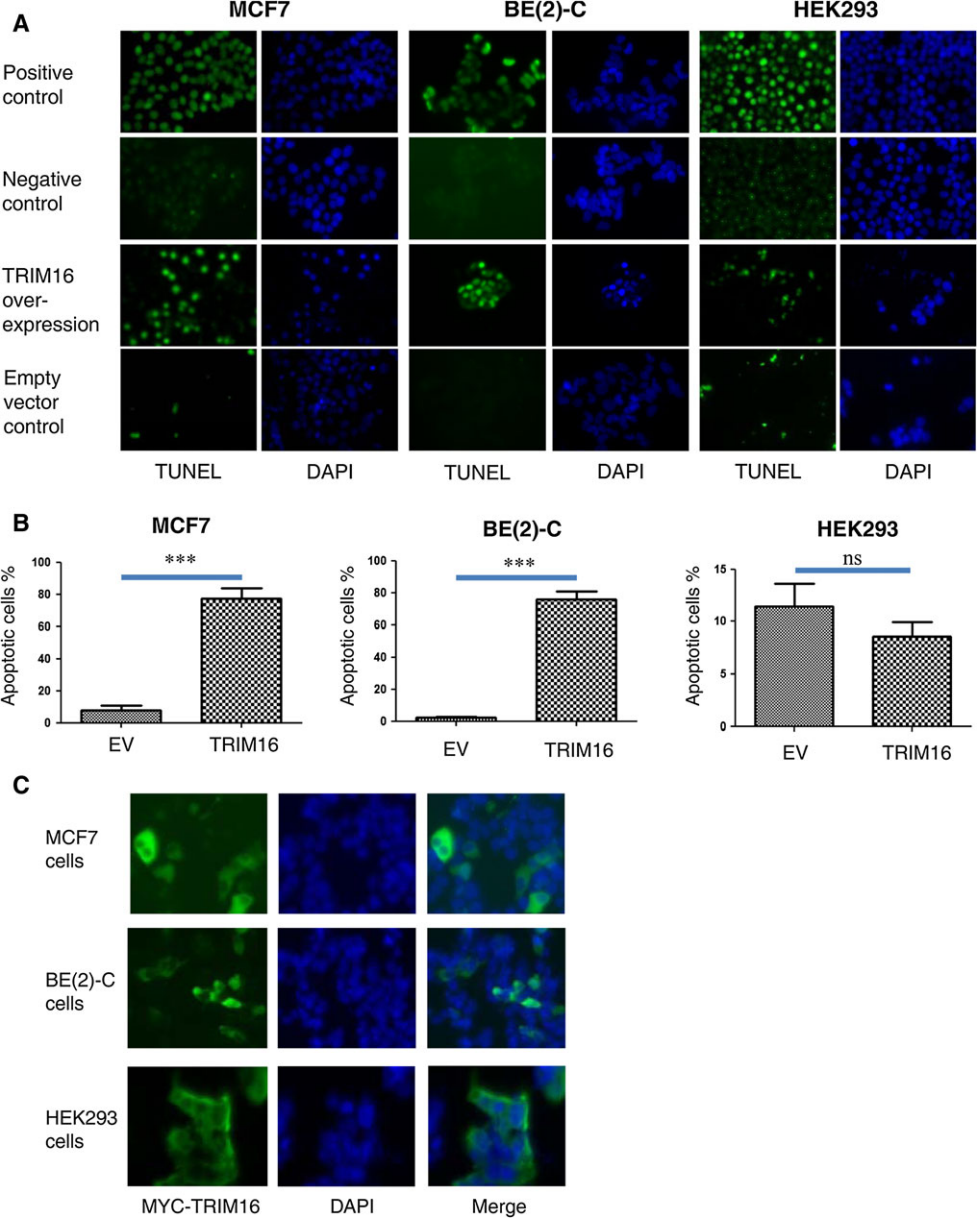
TRIM16 induces apoptosis in breast and neuroblastoma cancer cells. **a** Apoptosis was detected in MCF7 and BE(2)-C cancer cells overexpressing TRIM16 at 48 h post-transfection using the TUNEL TMR dye. No apoptosis was detected in the non-cancer HEK293 cells. **b** TUNEL TMR dye stained positive apoptotic cells were quantitated using fluorescence microscopy, 48 h post-transfection (***P < 0.001). **c** Expression of the transiently transfected MYC-tagged TRIM16 in the MCF7, BE(2)-C and HECK293 cells were detected by IF staining of the transfected TRIM16 protein (*green*) (Color figure online)

**Fig. 2:**
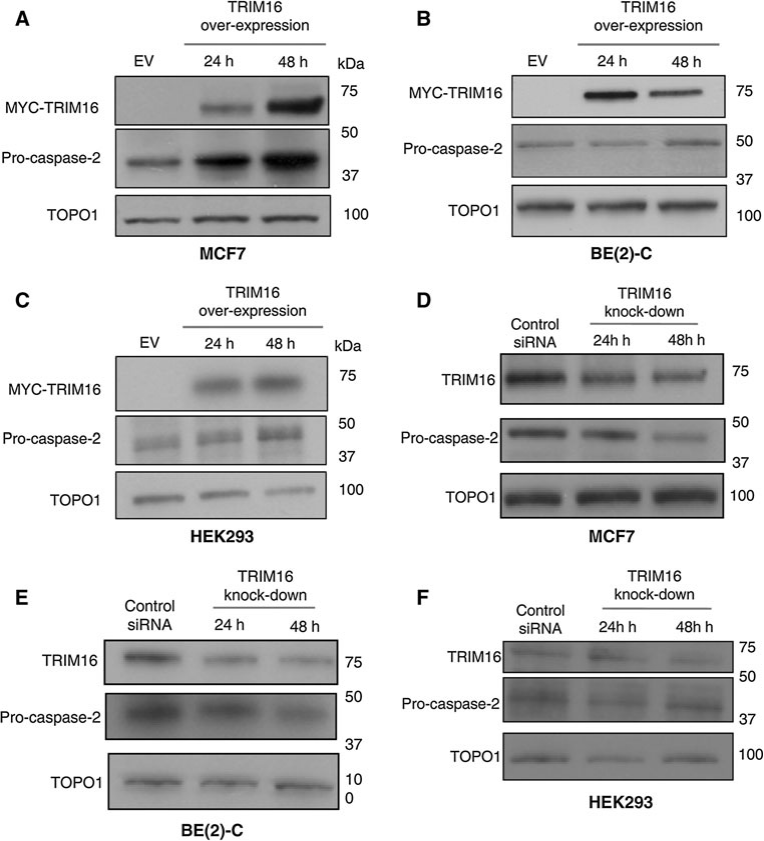
TRIM16 regulates procaspase-2 protein levels. **a–c** MCF7, BE(2)-C and HEK293 cells were transiently transfected with TRIM16 plasmid for 24 and 48 h. Western immunoblots of total protein extracts were analysed using antibodies specific to Myc-tag and caspase-2. Empty vector (EV) transfection was used as negative control. Topoisomerase-1 protein was used as the loading control. **d–f** TRIM16 protein expression was knock-downed in MCF7, BE(2)-C and HEK293 cells using specific siRNA to TRIM16. Total protein extracts were analysed with immunoblots using antibodies specific to TRIM16 and caspase-2. Control siRNA (siRNA) transfected cells were used as negative controls. Topoisomerase-1 was used as the loading control

**Fig. 3:**
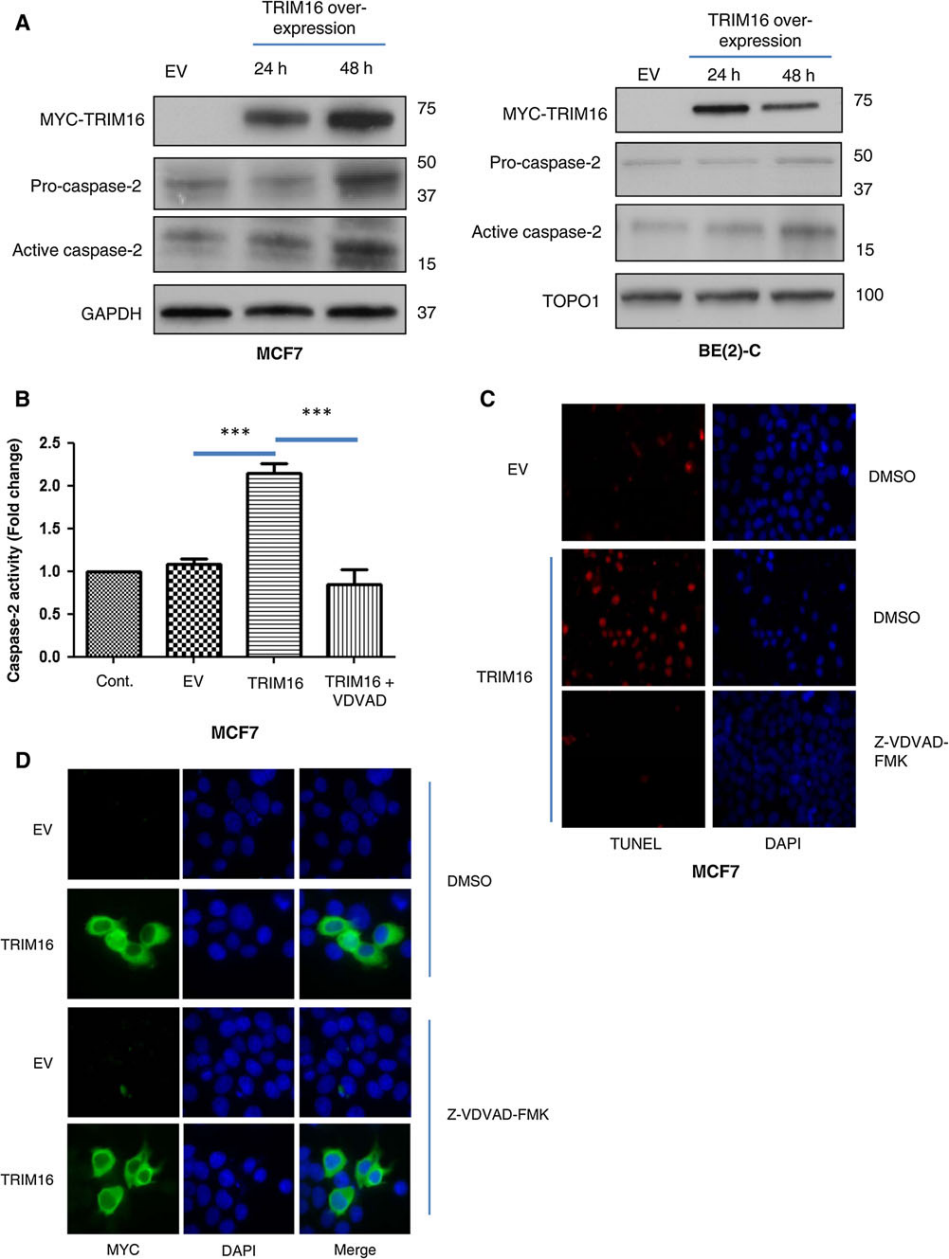
Chemical inhibition of caspase-2 suppresses TRIM16-induced caspase-2 activity and cell death. **a** Western immunoblot analysis of anti-procaspase-2 and caspase-2 active isoforms 24 and 48 h post transient transfection of TRIM16 in MCF7 and BE(2)-C cells. GAPDH was used as the loading control. **b** Caspase-2 activity levels at 24 and 48 h post TRIM16 transient transfection of MCF7 cells (***P < 0.001). **c** Apoptosis analysis by the TUNEL TMR dye of TRIM16 overexpressing MCF7 cells treated with the caspase-2 inhibitor VDVAD (40 μM). EV and TRIM16 transient transfected cells were used as negative and positive controls, respectively. **d** MYC-tagged transiently transfected MCF7 cells were detected by staining with MYC-tag antibody and fluorescent secondary antibody (*green*) (Color figure online)

We have previously shown increased apoptosis of MD-MBA-231 breast and SK-MES-1 lung cancer cells following forced expression of TRIM16 [14]. Here we first showed that apoptosis was a general feature of other cancer cell types, in this case MCF7 breast and BE(2)-C neuroblastoma cancer cell lines, following enforced overexpression of TRIM16 (Fig. 1a). Apoptotic cells were detected by TUNEL stain in MCF7 and BE(2)-C cells at 48 h after transient transfection of a TRIM16 expression vector, in contrast to the absence of any apoptotic cells in empty vector (EV) control cells (Fig. 1a). Quantitative analysis of the TUNEL images indicated an almost 80 % (*P* < 0.001) increase in apoptotic cells in the TRIM16 overexpressing MCF7 and BE(2)-C cells compared to the EV control samples (Fig. 2b). In comparison, non-malignant HEK293 cells transfected with TRIM16 demonstrated negligible induction of apoptosis (Fig. 1a, b). Efficiency of the MYC-tagged TRIM16 plasmid DNA was measured by staining the transiently transfected MCF7, BE(2)-C and HEK293 cells with Alexa FluorR 488 fluorescent antibody (Fig. 3c). Quantitation of the TRIM16 transfected cells indicated that approximately 60 % of the MCF7, BE(2)-C and HEK293 cells were transfected.

### TRIM16 regulates procaspase-2 protein levels

Caspases are the major proteins which initiates, regulates and executes apoptosis. We have previously performed cDNA microarray analysis and found that TRIM16 overexpression resulted in an increase in caspase-2 expression in neuroblastoma cancer cells. To investigate this further, TRIM16 was modulated in MCF7, BE(2)-C and HEK293 cells using transfection and siRNA technology. TRIM16 overexpression in MCF7, BE(2)-C and HEK293 cells resulted in an increased procaspase-2 protein levels in MCF7 cells at both 24 and 48 h time-points post-transfection, compared to EV controls. However, the increase in procaspase-2 was only observed in the MCF7 cells, but not in BE(2)-C and HEK293 cells (Fig. 2a–c). Conversely, inhibition of TRIM16 protein expression by a specific siRNA lowered expression levels of procaspase-2. A significant decrease in procaspase-2 expression was observed in the MCF7 cells at 24 and 48 h post-TRIM16 knock-down compared to the control siRNA (Fig. 2d). Similar decrease in procaspase-2 expression was also observed in BE(2)-C cells after siRNA inhibition of TRIM16 expression (Fig. 2e), albeit to a lesser extent. As expected knock-down of TRIM16 expression had no effect on the protein levels of procaspase-2 in the HEK293 cells (Fig. 2f). Together, these results suggest that TRIM16 can induce apoptotic cell death through modulating procaspase-2 levels.

### Chemical inhibition of caspase-2 suppresses TRIM16-induced caspase-2 activity and cell death

To better elucidate the effects of TRIM16 on caspase-2, we investigated caspase-2 activation using immunoblot analysis. Protein lysates from MCF7 and BE(2)-C cells overexpressing TRIM16 were immunoblotted with an antibody which recognises the activated or cleaved form of caspase-2. In Fig. 3a forced overexpression of TRIM16 caused a two fold increase (*P* ≤ 0.001) p value in the active form of caspase-2 at 48 h post-transfection. These regulatory effects of TRIM16 on caspase-2 protein levels were greatest in MCF7 cells, compared with BE(2)-C cells (Fig. 3a). Thus, further characterisation of the molecular functions of TRIM16 on caspase-2 was conducted mostly in the MCF7 cancer cells. To examine the role of TRIM16 in regulating caspase-2 activity, we measured caspase-2 activity by colorimetric assay. Activity assays on the MCF7 whole cell lysates of cells transiently transfected with TRIM16, showed more than a two-fold (*P* < 0.001) increase at 48 h, while no significant changes were detected in EV control cells (Fig. 3b). These data show a correlation between increased caspase-2 activities and increased levels of active caspase-2 protein. When the caspase-2 inhibitor, VDVAD (20 μM), was added to the whole cell lysates, caspase-2 activity was not detected (Fig. 3b). To determine whether TRIM16-induced activation of caspase-2 activity is required for the initiation of apoptosis, the TRIM16 overexpressing MCF7 cells were treated with the caspase-2 inhibitor VDVAD (40 μM) (Fig. 3c). These cells remained viable as determined by the lack of TUNEL staining of the cells and comparison with the staining observed in non-treated control cells (Fig. 3c). To confirm the transient transfect of MYC-tagged TRIM16 in the MCF7 cells, the cells were stained with MYC-tag antibody and fluorescent secondary antibody. Immunofluorescent images show that the MCF7 cells were transfected with MYC-tagged TRIM16 (Fig. 3d). The results further suggest that TRIM16 has a role in the activation process of caspase-2 and that activity of this caspase appears crucial for TRIM16-mediated apoptosis.

### TRIM16 directly interacts, and co-localises, with caspase-2

**Fig. 4:**
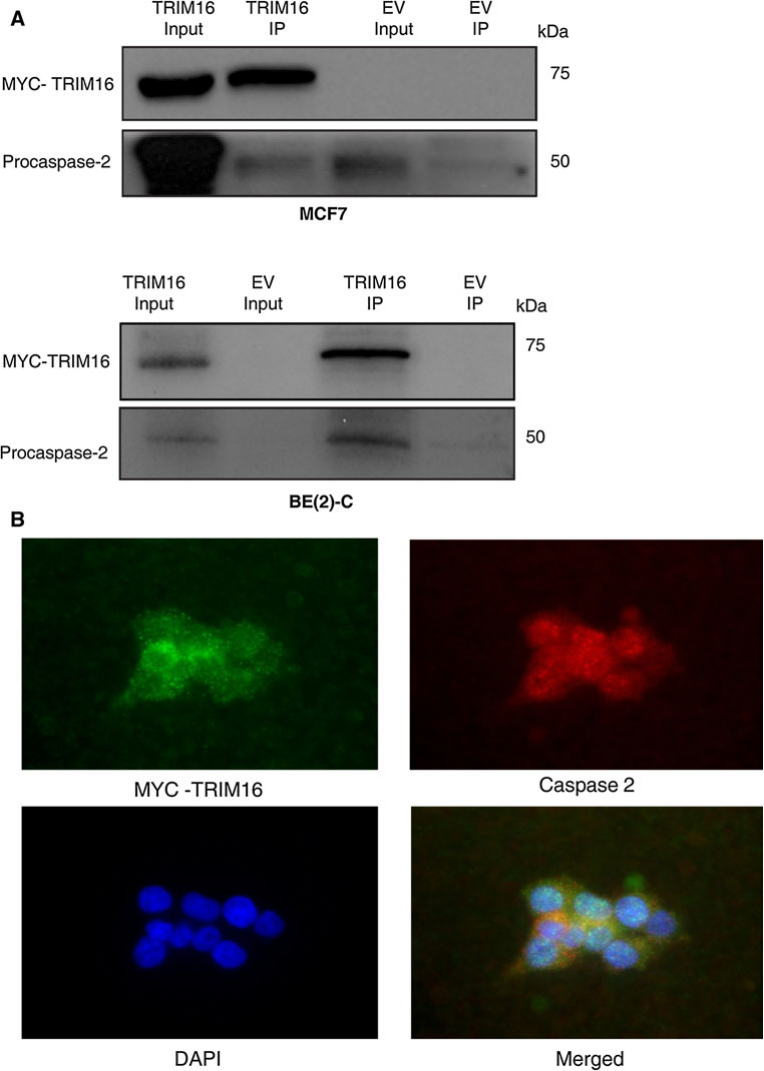
TRIM16 directly interacts, and co-localises, with caspase-2. **a** Total protein extracts from MCF7 and BE(2)-C cells transiently transfected with TRIM16 (TRIM16 input lane) or EV control (EV input lane) were incubated with His-specific magnetic beads. Purified immunoprecipitated complexes were analysed by Western blot with antibodies specific to MYC (TRIM16) and caspase-2. **b** Immunofluorescent confocal microscopy images of TRIM16-Myc/His transiently transfected MCF7 cells after transfection 48 h. TRIM16 protein was detected with MYC antibody and secondary Alexa Flour 488 antibody (*green*). Caspase-2 was detected with caspase-2 specific antibody and secondary Alexa Flour 555 (*red*) antibody, nuclear was staining using DAPI (*blue*) (Color figure online)

Our data suggested that TRIM16 induces apoptotic cell death through modulating procaspase-2. Since TRIM proteins are known to regulate cellular mechanisms through protein–protein interactions [4], thus, we examined whether there was a direct interaction of these proteins by co-IP and IF microscopy. Direct interaction of TRIM16 and caspase-2 in MCF7 and BE(2)-C cells was analysed by using His-tag specific magnetic beads. Protein lysates from cells transiently transfected with a TRIM16-Myc/His tagged expression vector were incubated with the His-tag-specific magnetic beads. Protein complexes that formed with TRIM16 were then analysed by Western immunoblot analysis. Detection of TRIM16-Myc/His with the Myc-tag-specific antibody found that TRIM16 protein was specifically pulled-down with the magnetic beads in both MCF7 and BE(2)-C cells, indicating that caspase-2 bound directly to TRIM16 and was precipitated in the complex with the magnetic beads (Fig. 4a). In order to determine the localisation of the TRIM16 and caspase-2 interaction, transiently transfected MCF7 cells were analysed by indirect IF confocal microscopy using antibodies to myc-TRIM16 (green) and caspase-2 (red). The results showed that TRIM16 is predominantly located in the cytoplasm, while caspase-2 is expressed in both the cytoplasm and the nucleus. However, the two proteins were found to co-localise in the cytoplasm of MCF7 cells (Fig. 4b).

### TRIM16 overexpression induces depolarisation of the mitochondrial membrane potential and cytochrome *c* release

**Fig. 5:**
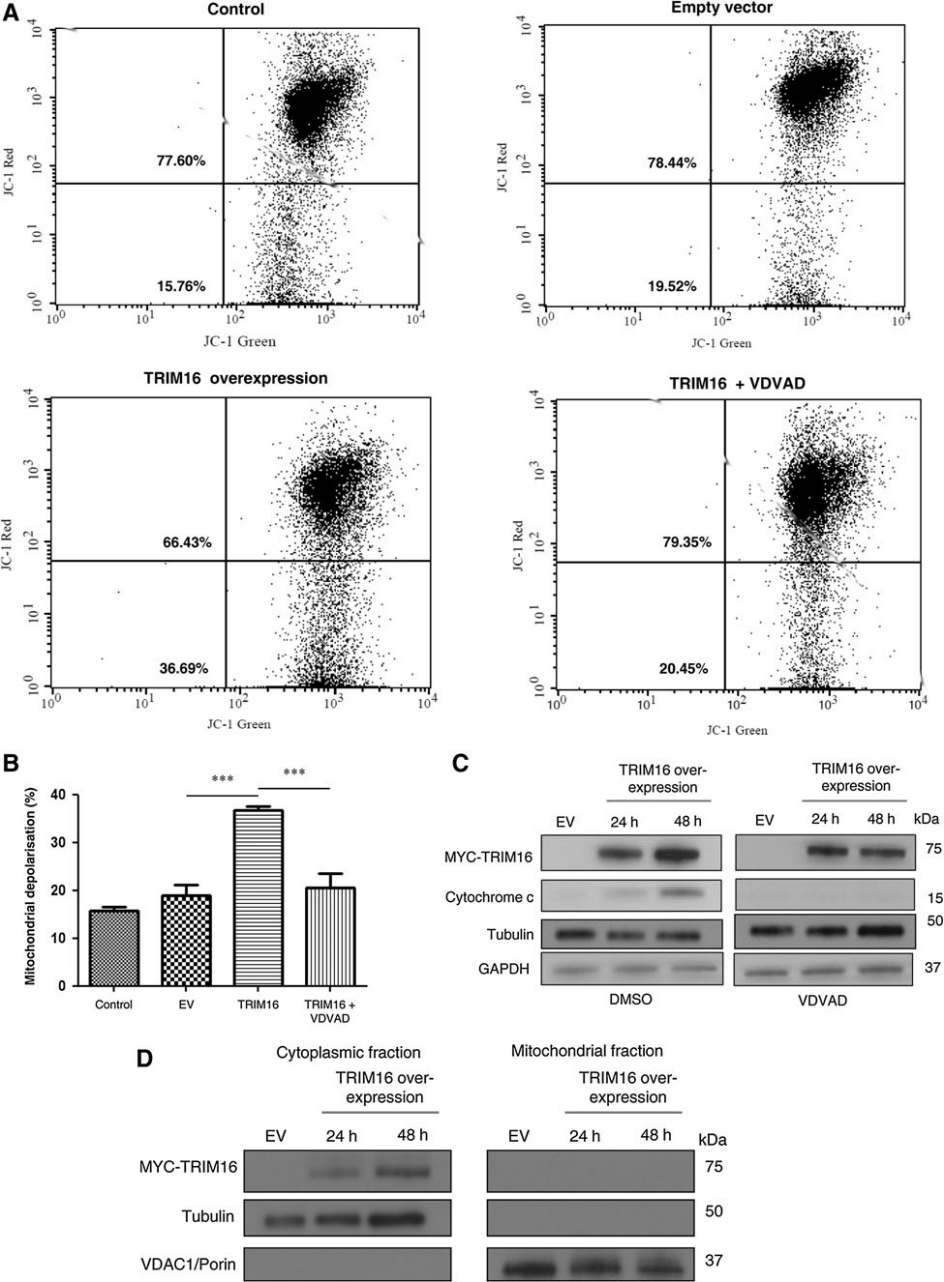
TRIM16 overexpression induces depolarisation of mitochondrial membrane potential and cytochrome *c* release. **a, b** Flow cytometry analysis for mitochondrial membrane depolarisation was performed in MCF7 cells transiently transfected with EV or TRIM16 (***P < 0.001). TRIM16 overexpressing samples treated with caspase-2 inhibitor VDVAD were also analysed by flow cytometry (*** *P* < 0.001). Non-treated MCF7 cells were used as the negative control. **c** The cytoplasmic extracts of TRIM16 transiently transfected MCF7 cells, treated with either DMSO or VDVAD were isolated and analysed by immunoblot using cytochrome *c* antibody. Tubulin was used a cytoplasmic protein loading control and GAPDH was used as the total protein loading control. Myc-tag antibody was used as for confirmation of TRIM16 plasmid transfection. **d** Fractionation of the cytoplasmic and mitochondrial proteins from the TRIM16 transiently transfected MCF7 cells were confirmed by using the cytoplasmic protein marker tubulin and the mitochondrial protein marker VDAC1/Porin

Caspase-2 has been previously reported to activate an intrinsic apoptotic pathway, dependent on the mitochondria [17, 18]. Thus, mitochondrial membrane potential was examined by flow cytometry after the overexpression of TRIM16 in MCF7 using MitoProbe™ JC-1. A significant increase in the rate of mitochondrial depolarisation of 40 % (*P* < 0.001) was observed following the activation of caspase-2 by the overexpression of TRIM16 (Fig. 5a, b). Depolarisation of mitochondrial membrane potential occurred at 48 h post-TRIM16 overexpression, while minimal depolarisation was detected at 24 h (data not shown). Inhibition of caspase-2 activity by the caspase-2 inhibitor VDVAD (40 μM) prevented this depolarisation (Fig. 5a, b).

Cytochrome *c* release into the cytoplasm is a common event following mitochondrial depolarisation, and several reports indicate activation of caspase-2 lies upstream of mitochondrial depolarisation [17, 18]. Therefore, the isolated cytoplasmic extracts from TRIM16 overexpressing MCF7 cells were analysed by immunoblot using antibodies to the Myc-tagged TRIM16 or cytochrome *c*. As anticipated, increasing amounts of cytochrome *c* were detected in the cytoplasmic extracts of TRIM16 overexpressing MCF7 cells over 48 h (Fig. 5c). Moreover, inhibition of caspase-2 activity by the caspase-2 inhibitor VDVAD in cells transient transfected with TRIM16 prevented the mitochondrial release of cytochrome *c* into the cytoplasm (Fig. 5c). Fractionation of the cytoplasmic and mitochondrial proteins were confirmed by using the tubulin and VDAC1/Porin antibodies as cytoplasmic and mitochondrial protein markers, respectively (Fig. 5d).

**Fig. 6:**
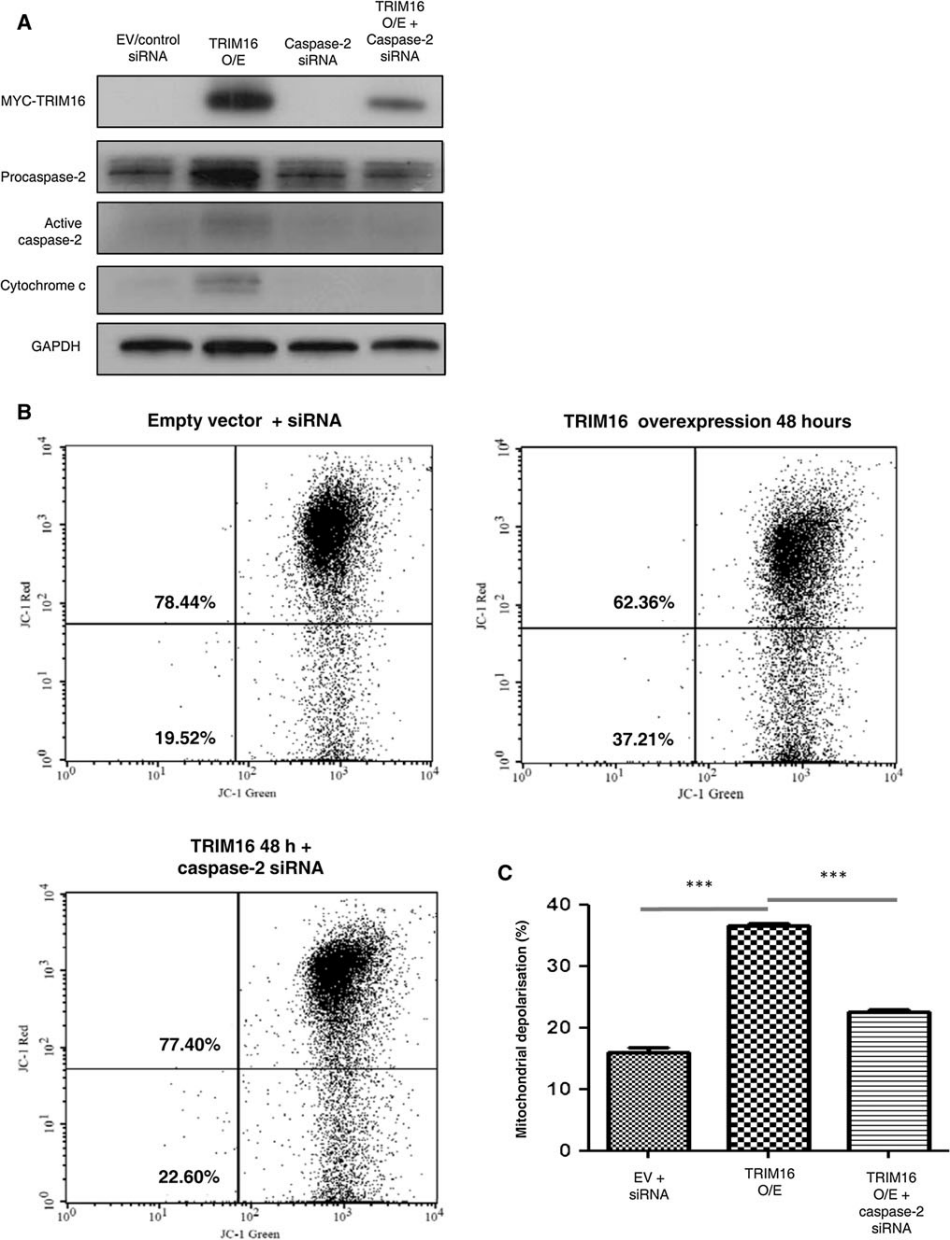
Caspase-2 activation, release of cytochrome *c* and mitochondrial depolarisation are required for TRIM16 induced apoptosis. **a** MCF7 cells were transiently transfected with EV control, TRIM16 plasmid, siRNA specific to caspase-2 or a combination of TRIM16 plasmid and caspase-2 siRNA for 48 h and analysed by immunoblotting with antibodies specific to MYC-TRIM16, caspase-2 and cytochrome *c*. GAPDH protein was used as the loading control. **b, c** Flow cytometry analysis for mitochondrial membrane depolarisation of MCF7 cells transfected with EV/siRNA control, TRIM16 plasmid and TRIM16 plasmid plus siRNA specific to caspase-2 was performed by using the MitoProbeTM JC-1 assay kit (***P < 0.001)

Although caspase-2 is the most specific caspase that is uniquely inhibited by VDVAD, the validity of the data obtained with this inhibitor were further confirmed by the use of caspase-2 specific siRNA to knock-down caspase-2 protein expression. Comparison of the procaspase-2 levels between the cells overexpressing TRIM16 alone and cells transfected with both TRIM16 expression vector and procaspase-2 siRNA shows significant decreases in pro- and active caspase-2 protein levels (Fig. 6a). Inhibition of caspase-2 expression also prevented the mitochondrial release of cytochrome *c*, in the TRIM16 overexpressing MCF7 cells (Fig. 6a). Furthermore, mitochondrial depolarisation was also prevented by the siRNA inhibition of caspase-2 expression (Fig. 6b, c). These data reveal that inhibiting caspase-2 protein expression by siRNA knock-down has same effects on apoptosis as the caspase-2 inhibitor VDVAD. Thus, TRIM16 requires caspase-2 activity for mitochondrial depolarisation and the induction of apoptosis.

## Discussion

Regulatory proteins which have been associated with programmed cell death are under intense investigation as potential cancer therapeutic targets. Determining the cellular processes which lead to activation of caspases during apoptosis, and the relevant intracellular caspase substrates and regulators, are a vital link in the pathway toward potential therapies [17]. Execution of apoptosis requires the accurate co-ordination of the cell death-specific proteases, the caspase protein family. Using a biochemical approach, our data presented in this study describes a novel mechanism of TRIM16-induced apoptosis that requires the activation and enzymatic activity of caspase-2.

Recently, the multifunctional cellular protein, TRIM16, has been shown to inhibit the growth and proliferation of several different human cancer cell lines [11–14]. These studies describe many regulatory roles of TRIM16, one of which involves E2F1 protein degradation that causes growth inhibition of cancer cells [12, 13]. Here we have shown that TRIM16 upregulates caspase-2 protein levels in breast cancer and neuroblastoma cells but not non-malignant cells. It is possible that as a result of increased levels of TRIM16, procaspase-2 increases and reaches a threshold for its activation, which initiates apoptosis.

Caspase-2 is an enigmatic caspase with active roles in both cell cycle regulation and programmed cell death [19, 20]. The enzyme also has the physiochemical properties of both an initiator and effector caspase [21]. Caspase-2 knock-out mice develop normally and are viable, while the absence of caspase-2 expression in the presence of an oncogenic signal led to the formation of aggressive tumours in immunodeficient mice [22], suggesting that this particular caspase is a tumour suppressor protein [22]. It has been shown that dimerization and cleavage are essential for functional activation of caspase-2 in apoptosis [23–25], and there are two known mechanisms of caspase-2 activation, and each mechanism involves different regulatory proteins. An activation platform known as the PIDDosome, is a trimolecular complex consisting of PIDD (a p53-induced protein with a death domain), RAIDD (receptor-interacting protein-associated ICH1/CED3 homologous protein with a death domain) and procaspase-2 [25]. Procaspase-2 proteins recruited to this activation platform and then dimerise. Upon dimerization, autocatalytic cleavage occurs and activated forms of caspase-2 are generated [23, 24, 26].

Another caspase-2 activation platform is instigated by the formation of a large multi-protein complex known as the death-inducing signalling complex (DISC). DISC formation requires up-regulation of CD95 by p53 and the recruitment of FADD and procaspase-8 [27–29]. Upon formation of the DISC complex, caspase-8 cleaves procaspase-2 resulting in activation of this caspase [28]. Unlike the PIDDosome, the interaction of procaspase-2 with the DISC complex occurs transiently [28]. As enforced overexpression of TRIM16 increases caspase-2 activity by more than 2-fold, it is highly plausible that TRIM16 may have a novel role in either of these activation platforms. It is possible that TRIM16 mediates the recruitment of caspase-2 to a platform to initiate the dimerisation and cleavage process. Similarly to the direct mediatory roles of TRIM16 in the pRb/E2F1 in cell cycle control pathway, TRIM16 may also be directly involved in caspase-2 regulation and activation for the initiation of apoptosis. The data obtained from MCF7 cancer cells suggested that all the cell death effects of TRIM16 overexpression required the integrity as well as the functional activation of caspase-2.

Almost all of the TRIM proteins regulate cellular function through protein–protein interactions. Inactive initiator caspases with long pro-domains contain specific protein–protein interaction motifs, which suggest these proteolytic enzymes are regulated prior to cleavage and subsequent activation [28, 30]. A physical interaction of TRIM16 with procaspase-2 in the cytoplasm of MCF7 cells suggests that TRIM16 may have a central role upstream of caspase-2 activation. Further studies are needed to provide evidence, and may prove that TRIM16 has a direct functional role in the recruitment and activation of caspase-2. This direct protein–protein interaction between TRIM16 and caspase-2 also suggests that TRIM16 may be involved in the PIDDosome caspase-2 activation platform, as the molecules in this complex are known to directly interact in the cytoplasm of cells [25].

Depolarisation of mitochondrial membrane potential results in the release of proapoptotic mitochondrial proteins, such as cytochrome *c* into the cytoplasm [31]. Caspase-2, through the cleavage of Bid activates Bax/Bak which form pores in the outer mitochondrial membrane allowing the release of proapoptotic proteins [29]. Upon release into the cytoplasm, cytochrome *c* activates the Apaf-caspase-9 apoptosome complex [17]. This mechanism lies downstream of caspase-2 activation and is required by caspase-2 to activate executioner caspases [17, 18]. Since TRIM16 has a central role involved in the activation process it is possible that, TRIM16 is a novel protein that has the potential to initiate the mitochondria-dependent intrinsic apoptosis pathway.

Caspase-3, also known as the death enzyme, has a crucial role in the controlled execution of programmed cell death. Many mechanisms involved in the intrinsic and extrinsic pathways of apoptosis require the activation of caspase-3 [32]. However, caspase-3 was not identified to be up-regulated in the TRIM16 overexpression microarray analysis (unpublished data). In addition, caspase-3 is an executioner caspase that has downstream functions in apoptosis, while caspase-2 is an initiator caspase with upstream functions in apoptosis. Furthermore, due to a deletion, MCF7 breast cancer cells lack of functional capacity to express caspase-3 [33]. Despite the absence of this central death caspase, our data suggested that TRIM16 induces cell death of MCF7 cells through the activation of caspase-2. Activation of caspase-2 alone is insufficient to execute all the downstream processes necessary for apoptosis. It is possible that once activated by TRIM16, caspase-2 is able to execute programmed cell death through alternate pathways activating other executioner caspases, such as caspase-7 [34].

Caspase-2 is a crucial component of the TRIM16-induced apoptosis pathway in MCF7 cells. Our results suggest that TRIM16-mediated apoptosis and the mechanisms leading to cell death are dependent on the functional integrity of caspase-2. However, some studies have questioned that the specificity of the inhibitor VDVAD in caspase-2 inhibition [35]. Although caspase-2 is specifically targeted by VDVAD, caspases—3, 7 and 10 were shown to have only limited activity when exposed to this same substrate [36]. Hence, to further confirm the role of caspase-2 in TRIM16 induced apoptosis, caspase-2 protein expression was knocked-down by specific siRNA. Near identical effects are observed with the siRNA knock-down of caspase-2 and the use of the inhibitor. With reduced caspase-2 protein expression, no active isoforms of caspase-2 were detected. The mitochondrial depolarisation did not occur and no cytochrome *c* was released into the cytoplasm. Thus, inhibiting caspase-2 by either protein knock-down or pharmacological inhibition reveals that TRIM16 requires the activation and activity of caspase-2 to function as an apoptotic molecule.

Taken together, our data reveal a novel mechanism, by which cells overexpressing TRIM16 can induce apoptosis in cancer cells, but not in non-malignant cells. It may be due to repressed and deregulated TRIM16 protein expression in cancer cells, but not in normal cells. TRIM16 directly interacts, and co-localises, with caspase-2, resulting in the depolarisation of mitochondria and a corresponding cytochrome *c* release. This mechanism and pathway is specific to cancer cells, where TRIM16 expression is often reduced and further supports the role of TRIM16 as a tumour suppressor protein.
